# Facile Synthesis of SnO_2_ Aerogel/Reduced Graphene Oxide Nanocomposites via in Situ Annealing for the Photocatalytic Degradation of Methyl Orange

**DOI:** 10.3390/nano9030358

**Published:** 2019-03-04

**Authors:** Taehee Kim, Vinayak G. Parale, Hae-Noo-Ree Jung, Younghun Kim, Zied Driss, Dorra Driss, Abdallah Bouabidi, Souhir Euchy, Hyung-Ho Park

**Affiliations:** 1 Department of Materials Science and Engineering, Yonsei University, Seoul 03722, Korea; taehee-kim@yonsei.ac.kr (T.K.); vinayakparale3@gmail.com (V.G.P.); nuri_j@yonsei.ac.kr (H.-N.-R.J.); younghun_kim@yonsei.ac.kr (Y.K.); 2 Laboratory of Electromechanical Systems (LASEM), National School of Engineers of Sfax (ENIS), University of Sfax, PO Box 1173, Route Soukra km 3.5, 3038 Sfax, Tunisia; zied.driss@enis.tn (Z.D.); bouabidi_abdallah@yahoo.fr (A.B.); echi.souhir@yahoo.com (S.E.); 3 Laboratory of Molecular and Cellular Screening Processes, Centre of Biotechnology of Sfax (CBS), University of Sfax, PO Box 1177, Road Sidi Mansour km 6, 3018 Sfax, Tunisia; dorra_driss@yahoo.fr

**Keywords:** SnO_2_ aerogel, sol–gel method, graphene oxide, nanocomposite, photocatalysis

## Abstract

SnO_2_ aerogel/reduced graphene oxide (rGO) nanocomposites were synthesized using the sol–gel method. A homogeneous dispersion of graphene oxide (GO) flakes in a tin precursor solution was captured in a three-dimensional network SnO_2_ aerogel matrix and successively underwent supercritical alcohol drying followed by the in situ thermal reduction of GO, resulting in SnO_2_ aerogel/rGO nanocomposites. The chemical interaction between aerogel matrix and GO functional groups was confirmed by a peak shift in the Fourier transform infrared spectra and a change in the optical bandgap of the diffuse reflectance spectra. The role of rGO in 3D aerogel structure was studied in terms of photocatalytic activity with detailed mechanism of the enhancement such as electron transfer between the GO and SnO_2_. In addition, the photocatalytic activity of these nanocomposites in the methyl orange degradation varied depending on the amount of rGO loading in the SnO_2_ aerogel matrix; an appropriate amount of rGO was required for the highest enhancement in the photocatalytic activity of the SnO_2_ aerogel. The proposed nanocomposites could be a useful solution against water pollutants.

## 1. Introduction

The demand for solutions against several environmental issues has recently increased; in particular, the presence of air pollutants and organic dyes in water are global concerns. Since the latters are often highly toxic and have mutagenic properties, their removal is a major problem in the industry [1,2,3]. Until now, many efforts have been made to decompose the organic pollutants by using various processes involving, e.g., (homogeneous and/or heterogeneous) catalysts, adsorbents, and ozone [4,5,6,7,8] and photocatalysis is one of the most effective and economical paths for their removal [9,10,11,12,13]. Semiconducting metal oxides such as TiO_2_, ZnO, and SnO_2_ have been widely used as photocatalysts due to their ability to generate electron–hole pairs when photon energy is provided [12,13,14,15,16,17,18,19]. Among them, SnO_2_ has gained much attention because of its high natural abundance, optical transparency, and physicochemical stability, relatively high electrical conductivity, and lack of toxicity [20,21,22]. SnO_2_ is an n-type semiconductor with a wide bandgap (3.6 eV) and a rutile-type crystal structure, but it exhibits a low photocatalytic activity due to such wide bandgap and its high photogenerated electron–hole pair recombination rate [23]. Also, the economic aspects of the preparation of SnO_2_ photocatalyst for the mass production is still challenging. The cost and compatibility should be considered in photocatalytic degradation [24,25,26,27,28].

Many previous studies aimed to enhance the photocatalytic activity of SnO_2_-based semiconductors by introducing nanostructures and composites [29,30,31,32,33]. Different nanostructures for SnO_2_-based photocatalysts, such as nanoparticles [29], flower-like structures [30], and simonkolleite nanopetals [31], have been reported so far. In addition, the use of SnO_2_ composites with carbon materials (in particular, carbon core-shell particles [34], graphene oxide (GO) [35], activated carbon [36], and fullerene [37]) instead of pure SnO_2_ to decrease the electron–hole recombination rate, which would enhance the photocatalytic activity, has been investigated. Moreover, some studies have reported chemical interactions between these carbon materials and the SnO_2_ matrix, which decrease the bandgap and improve the photocatalytic activity [38,39].

In the present work, we synthesized porous SnO_2_ aerogel/reduced GO (rGO) nanocomposites via an epoxide-assisted sol–gel process. GO flakes were uniformly dispersed in a tin solution and captured in a colloidal SnO_2_ three-dimensional (3D) network. Then, the in situ thermal reduction of GO was performed continuously in autoclave during the supercritical drying of the resulting nanocomposites. To the best of our knowledge, this is the first report on the in situ annealing of SnO_2_ aerogel/GO nanocomposites in an autoclave, providing SnO_2_ aerogel/rGO nanocomposites as the final product. Furthermore, the dispersion of 2D GO sheets would hinder the network formation. In this study, the reaction condition was carefully controlled to achieve the uniform distribution as well as the reaction between oxygen containing functional groups on GO sheet and Sn metal center and confirmed by various characterizations. In addition, the synthesized nanocomposites were analyzed in detail and their performance in the photocatalytic degradation of methyl orange (MO) was quantified. As per our knowledge, SnO_2_ aerogel and rGO composite has not been studied in application of photocatalysis.

## 2. Materials and Methods 

### 2.1. Sample Preparation

Tin tetrachloride pentahydrate (SnCl_4_·5H_2_O) was added to a mixture of water and ethanol (3:1, v/v) and stirred for 1 h; after complete dissolution, 0.05, 0.1, and 0.3 wt.% of GO flakes were added to each solution separately and their uniform distribution was ensured via ultrasonic processing for 1 h. When the GO flakes were suspended in the tin salt solution, this was chilled in an ice bath. Excess propylene oxide (C_3_H_6_O, 143 mmol; Sigma-Aldrich, St. Louis, MO, USA) was added to the solution dropwise by using a syringe. The chemistry of the epoxide-initiated gelation method is well described in [40]; the gelation took place in 10 min and the alcogel was left for 20 min to complete the reaction. The resulting alcogel was added with ethanol and aged at room temperature for 3 days by exchanging with fresh ethanol every 24 h. Then, the solvent exchange was performed with methanol for 24 h. The so-obtained SnO_2_ alcogel underwent supercritical methanol drying at 265 °C and 105 bar in an autoclave equipped with a 2 L vessel (Parr Instruments, Moline, IL, USA). After complete supercritical fluid extraction, in situ drying was performed in the same autoclave by heating the vessel to 300 °C under N_2_ flow and the dried aerogel was annealed for 1 h to induce the reduction of GO to rGO.

### 2.2. Characterization

A Fourier transform infrared spectroscopy (FTIR) system (Perkin Elmer, Waltham, MA, USA) was used to monitor the reaction and characterize the impurities. The specific surface area of nanoporous aerogel composites was measured using a Brunauer–Emmett–Teller (BET) analyzer (Quantachrome, Boynton Beach, FL, USA) and the Barrett–Joyner–Halenda (BJH) method. Their crystallinity and structure were investigated with an X-ray diffraction (XRD) system (Rigaku Ultima, Tokyo, Japan) using Cu Kα radiation (1.5418 Å) in the 20–80° 2θ range. The surface morphology of the SnO_2_ aerogel/rGO nanocomposites was analyzed using a field emission scanning electron microscopy (FESEM) system (JEOL JSM 7001F, Tokyo, Japan). The photoluminescence (PL) analysis was performed on a LabRam Aramis system (Horriba Jobin Yvon, Madrid, Spain) at room temperature and with a laser excitation wavelength of ~325 nm. Ultraviolet diffuse reflectance spectra (UV-DRS) were recorded on a spectrometer (JASCO 780, Tokyo, Japan) was performed using powder DRS kit at room temperature.

### 2.3. Photocatalyst Properties

The photocatalytic activity of both pristine SnO_2_ aerogel and SnO_2_ aerogel/rGO nanocomposites was determined based on the degradation degree of an aqueous MO dye solution. The photocatalytic decolorization of the MO solution was derived from its absorption (absorption peak at 464 nm by using a UV–visible (Vis) spectrophotometer (JASCO 570, Tokyo, Japan) in the 300–800 nm range. The initial concentration of the MO dye in the solution was 1 × 10^−5^ M and the photocatalyst (1 mg mL^−1^) was dispersed in it. The photocatalytic degradation was performed using a UV lamp (40 W; Philips TL-K) with peak intensity at 370 nm irradiating directly the solution after achieving the adsorption/desorption equilibrium for 30 min. The solution was stirred continuously with a magnetic stirrer. After centrifugation with a microcentrifuge (DAIHAN CF−10, Seoul, Korea), the absorbance spectra of the solution with the photocatalyst suspension (approximately 1.2 mL aliquots) were recorded over time at ambient conditions. 

## 3. Results and Discussion

**Figure 1:**
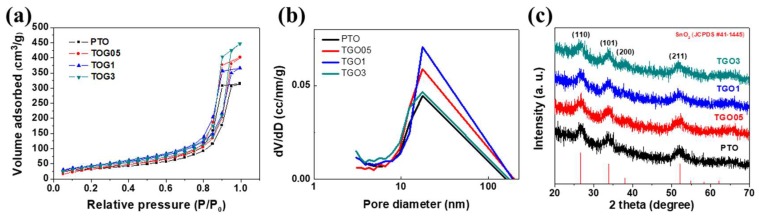
(**a**) Brunauer–Emmett–Teller isotherms, (**b**) Barrett–Joyner–Halenda graph and (**c**) X-ray diffraction spectra of pristine SnO_2_ aerogel (PTO) and SnO_2_ aerogel/reduced graphene oxide (rGO) nanocomposites with different rGO loadings (0.05 wt.%: TGO05; 0.1 wt.%: TGO1; 0.3 wt.%: TGO3).

Metal alkoxides are generally used as non-silica-based precursors in the synthesis of aerogels [41,42,43], but they are costly and their reactivity is hard to control [44]. In this study, cost effective tin chloride was selected as the precursor and propylene oxide was used to initialize the sol–gel process for the synthesis of the SnO_2_ aerogel/rGO nanocomposites. Propylene oxide acts as proton scavenger and ring-strained epoxide, increasing the hydrolysis and condensation rate [45]; its addition usually speeds up the gelation (less than a minute), leading to the formation of an opaque white alcogel [40,46]. However, in this study, the tin precursor solution and propylene oxide were chilled in an ice bath to obtain uniform pores in the SnO_2_ aerogel, giving a clear transparent alcogel. In addition, due to the relatively fast gelation (approximately 10 min), the GO flakes homogeneously dispersed via ultrasonication were trapped within the SnO_2_ matrix without large agglomerations. The porous SnO_2_ aerogel/rGO nanocomposites were obtained by removing the solvent in the 3D wet gel via drying at supercritical pressure and temperature. The thermal annealing method was used to reduce GO to rGO by decomposing the oxygen-containing functional groups. The BET and XRD results about the textural and crystalline properties of the as-synthesized pristine SnO_2_ aerogel (PTO) and SnO_2_ aerogel/rGO nanocomposites (named as TGO05, TGO1, and TGO3 according to the rGO loading of 0.05, 0.1, and 0.3 wt.%, respectively) are shown in Figure 1 and Table 1.

The nanoporous structure of the various samples was determined via the BET analysis (Figure 1a). All isotherms showed type V according to the IUPAC classification, revealing the mesoporous structure of the SnO_2_ aerogel. PTO exhibited a slightly smaller surface area compared to the rGO-added nanocomposites, while TGO3 showed the largest one (157 m^2^ g^−1^); the specific surface area of the SnO_2_ aerogel increased with the rGO loading. The rGO presence could clearly enhance this parameter without hindering the colloidal aerogel formation and this was due to the high initial surface area of the GO flakes. Moreover, the well-distributed rGO sheets with a high mechanical strength could reduce the thermal stress induced during the thermal annealing step. Moreover, the average pore volumes and pore sizes, calculated via the BJH method (Figure 1b), slightly increased in the SnO_2_ aerogel/rGO nanocomposites compared to the pristine sample. 

Figure 1c shows the XRD patterns of the SnO_2_ aerogel/rGO nanocomposite after the heat treatment. The SnO_2_aerogel exhibited a rutile-type tetragonal crystal structure and the XRD peaks at 26.61°, 33.89°, 37.95°, and 51.78° corresponded to the (110), (101), (200), and (211) crystal planes of the SnO_2_ aerogel/rGO nanocomposites, respectively, with the following cell parameters: a, b = 4.738 Å and c = 3.187 Å (JCPDS #41–1445) [47]. However, the graphene diffraction pattern could not be indexed because of the low amount of GO flakes. The average crystallite size of the aerogel nanocomposites, calculated using the Scherrer equation, was approximately 3.4 nm for all the samples and no distinctive influence of the rGO addition was observed on the crystallinity of the SnO_2_ aerogel.

Exfoliated GO can be reduced to rGO via simple annealing under oxidizing or inert atmosphere [48], but annealing SnO_2_ aerogel/GO composites under air atmosphere would reduce the number of oxygen vacancies in the aerogel, lowering its photocatalytic activity. Therefore, after drying, the reaction vessel was heated to 300 °C to induce the reduction of the GO flakes [48]; this is a facile method for in situ thermal annealing with desirable atmosphere consecutively just after the supercritical alcohol drying. For comparison, the BET specific surface areas of PTO and the various SnO_2_ aerogel/rGO nanocomposites were measured before, during, and after (i.e., after the supercritical drying) the in situ annealing (Table 2). The results suggested that the in situ annealing method can minimize the thermal stress. Therefore, the GO reduction in the SnO_2_ aerogel matrix via in situ annealing could introduce the enhancement of the specific surface area.

**Figure 2:**
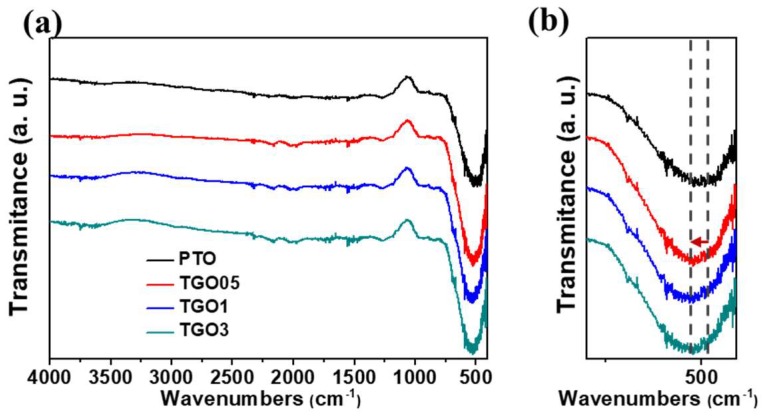
(**a**) Fourier transform infrared spectra and (**b**) their magnification in the low-wavenumber region for the pristine SnO_2_ aerogel (PTO) and the SnO_2_ aerogel/reduced graphene oxide (rGO) nanocomposites with different rGO loadings (0.05 wt.%: TGO05; 0.1 wt.%: TGO1; 0.3 wt.%: TGO3).

Figure 2 shows the FTIR spectra of PTO and the various SnO_2_ aerogel/rGO nanocomposites. All samples show weak and broad absorption peaks of hydroxyl (–OH) group around 3400 cm^−1^ owing to the surface hydroxyl groups on the SnO_2_ aerogel primary particles. This represents the hydrophilic surface which would favor to absorb aqueous solution for photocatalytic organic dye degradation [49]. In addition, Figure 2a shows a broad absorption peak at around 495 cm^−1^, for all samples, that indicates the formation of Sn–O–Sn bonds via the epoxide-initiated sol–gel process. However, this peak shifted toward higher wavenumbers with increasing the GO content, as shown in the magnified spectra in Figure 2b. This broad peak was probably a combination of Sn–O–Sn vibration and Sn–O–C stretching vibration (520 cm^−1^) [50,51]. The presence of the latter confirmed the chemical bond formation between SnO_2_ aerogel and residual oxygen-containing functional groups in GO during the synthesis.

**Figure 3:**
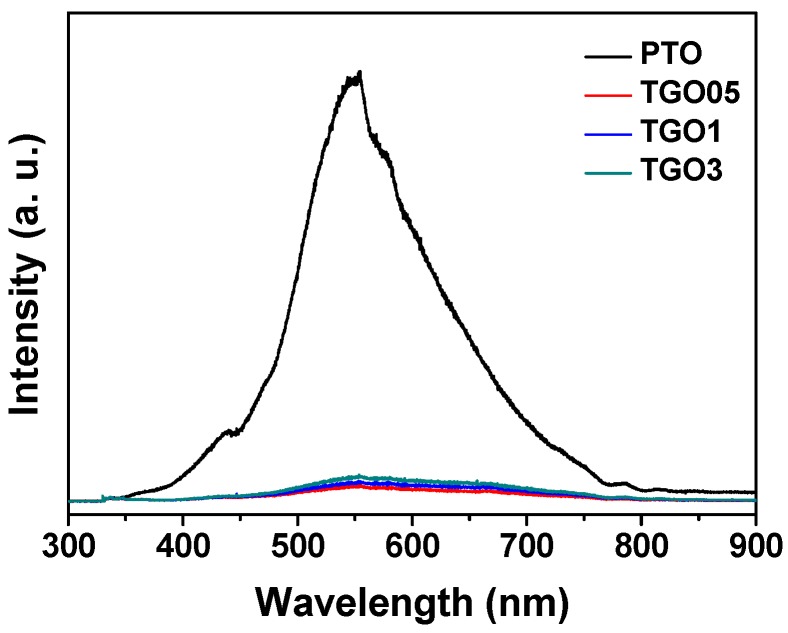
Photoluminescence spectra of pristine SnO_2_ aerogel (PTO) and SnO_2_ aerogel/reduced graphene oxide (rGO) nanocomposites with different rGO loadings (0.05 wt.%: TGO05; 0.1 wt.%: TGO1; 0.3 wt.%: TGO3).

PL emission is an important tool to determine the charge transportation and separation of electron–hole pairs. Figure 3 shows the photoluminescence spectra of PTO and the various SnO_2_ aerogel/rGO nanocomposites, measured at 325 nm. PTO exhibited a strong emission at around 554 nm due to the crystal defect of the SnO_2_ matrix [52], which significantly decreased in TGO05, TGO1, and TGO3; however, these samples showed similar intensities regardless the rGO amount added. This phenomenon indicated that the rGO addition could inhibit the electron–hole pair recombination, which implied that the rGO-based nanocomposites acted as electron trapping sites, benefiting the charge transfer in the enhanced photocatalytic activity. In addition, the PL quenching effect was also caused by the excellent electrical conductivity of rGO which will favor the photocatalytic activity [23].

**Figure 4:**
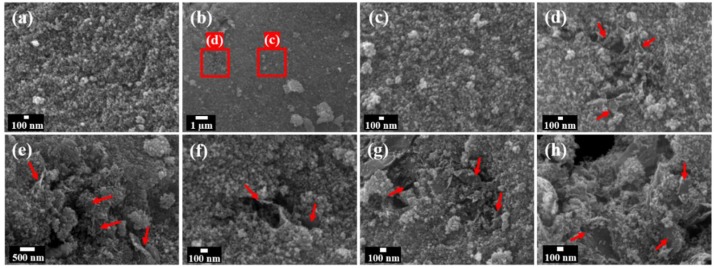
Field-emission scanning electron microscopy images of: (**a**) pristine SnO_2_ aerogel (PTO); (**b**) SnO_2_ aerogel/reduced graphene oxide (rGO) nanocomposites with an rGO loading of 0.1 wt.% (TGO1); (**c**,**d**) magnification of red square areas in (**b**), showing the rGO incorporation in the SnO_2_ aerogel matrix; (**e–h**). The red arrows represent rGO sheets.

The FESEM images of PTO and TGO1 are shown in Figure 4. In regards to the various rGO-added samples, only the results for TGO1 are presented because they all exhibited similar surface morphologies but more rGO graphene incorporation sites are found. The increase in the rGO loading did not influence the surface morphology of the nanocomposites. The colloidal porous nature of PTO was confirmed by a mesoporous aerogel network (Figure 4a). Figure 4d confirms the incorporation of rGO in the SnO_2_ matrix, which clearly depicts the growth of primary particles of aerogel on the surface of rGO. The visible well deposition of the aerogel particles allowed us to assume that there was a chemical interaction between the aerogel matrix and the functional groups on the rGO surface.

**Figure 5:**
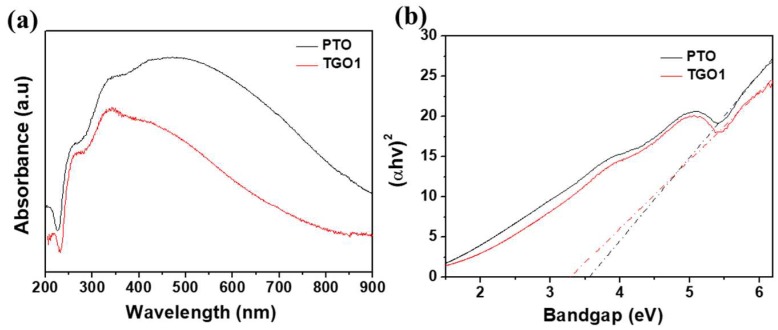
(**a**) Diffuse reflectance spectra and (**b**) Kubelka–Munk plots for the calculated bandgaps of pristine SnO_2_ aerogel (PTO) and SnO_2_ aerogel/reduced graphene oxide (rGO) nanocomposites with an rGO loading of 0.1 wt.% (TGO1).

The chemical bond formation between SnO_2_ matrix and the functional groups on GO was further confirmed by the UV-DRS analysis. Figure 5a shows the DRS spectra of PTO and TGO1 samples, revealing a considerable difference after the GO addition. Figure 5b shows their Kubelka–Munk plots; the calculated optical bandgaps of the semiconductor powders were approximately 3.56 and 3.29 eV for PTO and TGO1, respectively. The bandgap of PTO well agreed with the value for SnO_2_ nanoparticles reported in literature [53]. The rGO incorporation corresponded to a narrower bandgap, which could be attributed to the Sn–O–C bond formation between SnO_2_ and the rGO functional groups [50]. Since new impurity energy levels above the valence-band edge would be generated by the rGO introduction, a smaller input energy would be required for the charge carrier excitation. Therefore, the SnO_2_ aerogel/rGO nanocomposite exhibited a decrease in the optical bandgap with the Sn–O–C bond formation. Although this phenomenon has been previously observed in the case of TiO_2_-graphene composites, no such mechanism has been reported for SnO_2_-reduced graphene composites so far [54].

**Figure 6:**
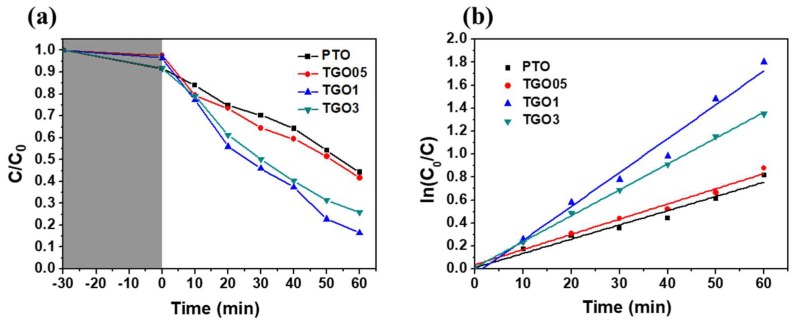
Photocatalytic activities of pristine SnO_2_ aerogel (PTO) and SnO_2_ aerogel/reduced graphene oxide (rGO) nanocomposites with different rGO loadings (0.05 wt.%: TGO05; 0.1 wt.%: TGO1; 0.3 wt.%: TGO3), represented as (**a**) the degradation of methyl orange (MO) dye (in terms of concentration ratio C/C_0_, where C_0_ and C are the MO concentrations at initial time t_0_ and a given ultraviolet irradiation time t) and (**b**) its reaction rate during time.

The photocatalytic activity of both pristine SnO_2_ aerogel and its rGO nanocomposites was investigated by the degradation of an MO solution. The MO concentration was determined by the absorbance of the solution at 464 nm using UV–Vis spectrophotometer after the centrifugal separation of the photocatalyst powder. All the MO solutions (with pristine and nanocomposite catalysts) were left in dark for 30 min to achieve the adsorption/desorption equilibrium state on the photocatalyst surface. Then, the photocatalytic activity of the samples was initiated via UV light irradiation under continuous magnetic stirring. The absorbance of the MO solution was measured starting from time t = 0, every 10 min of irradiation. Figure 6 shows the changes in the concentration ratio C/C_0_, where C_0_ and C are the MO concentrations at the initial time t_0_ and the irradiation time t, respectively, for the various samples. PTO exhibited a moderately reasonable photocatalytic activity, with a 56% MO degradation after 60 min of UV irradiation. Moreover, the rGO addition resulted in enhanced photocatalytic activity, reaching with TGO1 an 84% MO degradation in the same time. The activity of TGO05 was also relatively higher compared to PTO, but the difference was negligible. The enhancement in the photocatalytic activity of TGO1 was discussed by a comparison with PTO, confirming that the rGO introduction can considerably enhance the photocatalytic degradation of MO. 

Figure 6b shows the photocatalytic reaction rate constant k values, derived from the slope of the ln(C_0_/C) versus time plot, for PTO, TGO05, TGO1, and TGO3. All samples exhibited a first-order rate law with a linear behavior. The highest (2.9 × 10^−2^ min^−1^) and lowest (1.2 × 10^−2^ min^−1^) k values were observed with TGO1 and PTO, respectively, meaning that TGO1 attained a 2.4-fold higher photocatalytic degradation rate compared to PTO. 

**Figure 7:**
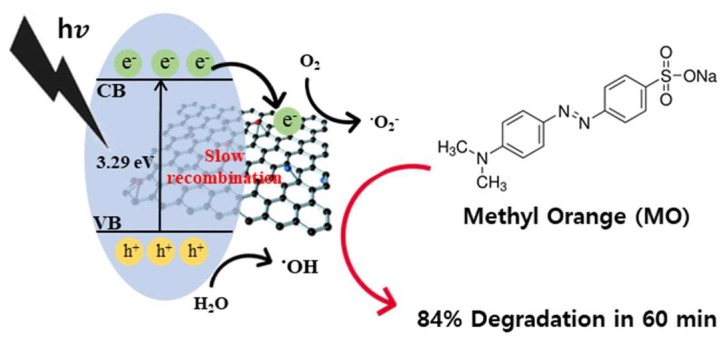
Schematic of MO dye photodegradation by using SnO_2_ aerogel/reduced graphene oxide nanocomposites under ultraviolet light.

In general, the three main factors influencing the photocatalytic activity are the light absorption intensity, the specific surface area, and the separation/recombination rate of photoexcited electron–hole pairs [23]. In this study, the rGO addition to SnO_2_ sol increased the specific surface area of the final aerogel nanocomposites by introducing more reactive surface sites and, hence, generating more electron–hole pairs [55]. In addition, the photoexcited electrons could move to the conduction band of rGO to interact with absorbed O_2_ and generate reactive radicals for the MO degradation; this phenomenon was hindered by the electron–hole pair recombination, enhancing the photocatalytic activity of the SnO_2_ aerogel/rGO nanocomposites. The detailed mechanisms for the formation of reactive radical intermediates during the photoactivation of the SnO_2_/rGO nanocomposites are described by the Equations (1)–(6) and the illustration in Figure 7. The photogenerated electron could move to rGO and produce reactive superoxide anions, eventually leading to the MO degradation.

SnO_2_ + hv → SnO_2_(e^−^ + h^+^) (1)

SnO_2_(e^−^ + h^+^) + rGO → SnO_2_(h^+^) + rGO(e^−^) (2)

SnO_2_(h^+^) + H_2_O → SnO_2_ + OH^·^ + H^+^ (3)

rGO(e^−^) + O_2_ → rGO + O_2_^·−^ (4)

O_2_^·−^ + H_2_O → H_2_O^·^ + OH^−^ (5)

MO + OH^·^/O_2_^·−^/H_2_O^·^ → CO_2_ + H_2_O (6)

On the other hand, the addition of excess graphene to the SnO_2_ aerogel matrix (i.e., the TGO3 case) reduced the enhancement of the photocatalytic activity, resulting in a 74% MO degradation in 60 min of UV irradiation. The photocatalytic activity was in the following order: TGO1 > TGO3 > TGO05 > PTO. Hence, a suitable amount of rGO loading would benefit the photocatalytic activity while its excess would decrease the photon absorption efficiency by SnO_2_ [56,57]; furthermore, such enhancement could be possible only at a certain extent. At the beginning state of the study, the experiment was designed to have 0.5–2.0 wt.% of rGO in SnO_2_ aerogel. However, authors have found that large amount of rGO in SnO_2_ aerogel retarded the photocatalytic activity as well as the surface area. This would happen because an excess rGO loading could increase the probability of collisions between photogenerated electrons and holes, favoring the electron–hole pair recombination and, thus affecting the photocatalytic activity [58]. Moreover, SnO_2_ aerogel/rGO nanocomposites are compared with previous reported SnO_2_ based photocatalysts in Table 3.

## 4. Conclusions

We introduced GO into an SnO_2_ aerogel matrix followed by its reduction to rGO via a facile in situ annealing process in a supercritical autoclave. During the sol–gel synthesis, the dispersed rGO flakes were captured in the SnO_2_ matrix and the colloidal SnO_2_ reacted with their functional groups, as confirmed by the FTIR and UV-DRS spectra. The so-obtained nanocomposites exhibited enhanced photocatalytic activity in the degradation of an MO dye solution by reducing the electron–hole pair recombination rate; the photogenerated electrons were trapped in rGO, leading to the formation of reactive superoxide anions, and the photocatalytic activity varied depending on the rGO loading in the SnO_2_ aerogel matrix. This enhancement in photocatalytic activity with rGO addition in aerogel structure was due to the movement of photogenerated electrons to conduction band in rGO which reduces the recombination rate. In 60 min under UV irradiation, the pristine SnO_2_ aerogel and the SnO_2_ aerogel/rGO (0.1 wt.%) nanocomposites achieved an MO degradation of 56% and 84% degradation, respectively. Therefore, SnO_2_ aerogel/rGO nanocomposites could be good candidates for the photodegradation of organic pollutants in industrial wastewater.

## Figures and Tables

**Table 1:** Textural properties and crystallite size of pristine SnO_2_ aerogel and SnO_2_ aerogel/reduced graphene oxide (rGO) nanocomposites with different rGO loadings.

Graphene Content (wt.%)	Surface Area (m^2^ g^−1^)	Pore Volume (cm^3^ g^−1^)	Pore diameter (nm)	Average Crystallite Size (nm)
0	117	0.49	17	3.48
0.05	131	0.62	19	3.42
0.1	147	0.69	18	3.46
0.3	157	0.57	15	3.35

**Table 2:** Comparison of the effect of in situ annealing on the specific surface area of SnO_2_ aerogel, with and without the addition of graphene oxide.

Graphene Content (wt.%)	Surface Area (m^2^ g^−1^) Before Annealing	Surface Area (m^2^ g^−1^) Facile, in-situ Annealing	Surface Area (m^2^ g^−1^) Post Annealing
0	199	117	84
0.05	277	131	75
0.1	314	147	73
0.3	359	157	92

**Table 3:** Comparison of photocatalytic performance of SnO_2_ aerogel/rGO nanocomposite photocatalyst in this work with previously reported composite photocatalysts.

Photocatalyst	Light Source/Pollutant	Experimental Condition	Photodegradation Efficiency	Ref.
SnO_2_ nanoparticles coated on MWCNT	4 × 6 W fluorescent halogen lamps (254 nm), methyl orange	C: 1000 mg L^−1^; P: 20 mg L^−1^	45 min/94%	[17]
Simonkolleite nanopetals with SnO_2_	3 × 8 W UV lamps (254 nm), rhodamine 6G	C: 500 mg L^−1^; P: 10 mg L^−1^	32 min /100%	[25]
SnO_2_–graphene nanocomposite (solid state)	300 W mercury lamp, methyl orang and rhodamine B	C: 500 mg L^−1^; P: 20 mg L^−1^	Methyl orange 40 min/95% Rhodamine B 60 min/97%	[49]
SnO_2_–CNT nanocomposites	9 W eight UV–vis lamps (365 nm), methylene blue and methyl orange	C: 200 mg L^−1^; P: 20 ppm (methylene blue), 10 ppm (methyl orange)	Methylene blue 180 min/93% Methyl orange 180 min/79%	[1]
SnO_2_ aerogel/rGO nanocomposite	40 W UV lamp (370 nm), methyl orange	C: 1 × 10^−5^ M; P: 100 mg L^−1^	Methyl orange 60 min/84%	This work

## References

[1] Kim S.P., Choi M.Y., Choi H.C. (2015). Characterization and photocatalytic performance of SnO_2_—CNT nanocomposites. Appl. Surf. Sci..

[2] Zhu X., An S., Liu Y., Hu J., Liu H., Tian C., Dai S., Yang X., Wang H., Abney C.W. (2017). Efficient removal of organic dye pollutants using covalent organic frameworks. Aiche J..

[3] Sreekanth T.V.M., Shim J.-J., Lee Y.R. (2017). Degradation of organic pollutants by bio-inspired rectangular and hexagonal titanium dioxide nanostructures. J. Photochem. Photobiol. B Biol..

[4] Wang W., Tadé M.O., Shao Z. (2015). Research progress of perovskite materials in photocatalysis- and photovoltaics-related energy conversion and environmental treatment. Chem. Soc. Rev..

[5] Upadhyay R.K., Soin N., Roy S.S. (2014). Role of graphene/metal oxide composites as photocatalysts, adsorbents and disinfectants in water treatment: A review. RSC Adv..

[6] Qin C., Li Z., Chen G., Zhao Y., Lin T. (2015). Fabrication and visible-light photocatalytic behavior of perovskite praseodymium ferrite porous nanotubes. J. Power Sour..

[7] Yang Z., Chen S., Fu K., Liu X., Li F., Du Y., Zhou P., Cheng Z. (2018). Highly efficient adsorbent for organic dyes based on a temperature- and pH-responsive multiblock polymer. J. Appl. Polym. Sci..

[8] Zucker I., Lester Y., Avisar D., Hübner U., Jekel M., Weinberger Y., Mamane H. (2015). Influence of Wastewater Particles on Ozone Degradation of Trace Organic Contaminants. Environ. Sci. Technol..

[9] Lee D.-S., Lee S.-Y., Rhee K.Y., Park S.-J. (2014). Effect of hydrothermal temperature on photocatalytic properties of TiO_2_ nanotubes. Curr. Appl. Phys..

[10] Pant A., Tanwar R., Kaur B., Mandal U.K. (2018). A magnetically recyclable photocatalyst with commendable dye degradation activity at ambient conditions. Sci. Rep..

[11] Wang M., You M., Zhang K., Zhang Y., Han J., Fu R., Liu S., Zhu T. (2018). Bi_3.64_Mo_0.36_O_6.55_/Bi_2_MoO_6_ heterostructure composite with enhanced photocatalytic activity for organic pollutants degradation. J. Alloy. Compd..

[12] Zhang Y., Park M., Kim H.-Y., El-Newehy M., Rhee K.Y., Park S.-J. (2015). Effect of TiO_2_ on photocatalytic activity of polyvinylpyrrolidone fabricated via electrospinning. Compos. Part B Eng..

[13] Wang H., Qiu X., Liu W., Yang D. (2017). Facile preparation of well-combined lignin-based carbon/ZnO hybrid composite with excellent photocatalytic activity. Appl. Surf. Sci..

[14] Anjum M., Miandad R., Waqas M., Gehany F., Barakat M.A. (2016). Remediation of wastewater using various nano-materials. Arab. J. Chem..

[15] Karri R.R., Tanzifi M., Tavakkoli Yaraki M., Sahu J.N. (2018). Optimization and modeling of methyl orange adsorption onto polyaniline nano-adsorbent through response surface methodology and differential evolution embedded neural network. J. Environ. Manag..

[16] Pastrana-Martínez L.M., Morales-Torres S., Likodimos V., Figueiredo J.L., Faria J.L., Falaras P., Silva A.M.T. (2012). Advanced nanostructured photocatalysts based on reduced graphene oxide–TiO_2_ composites for degradation of diphenhydramine pharmaceutical and methyl orange dye. Appl. Catal. B Environ..

[17] Khalid N.R., Ahmed E., Hong Z., Zhang Y., Ullah M., Ahmed M. (2013). Graphene modified Nd/TiO_2_ photocatalyst for methyl orange degradation under visible light irradiation. Ceram. Int..

[18] Filice S., D’Angelo D., Libertino S., Nicotera I., Kosma V., Privitera V., Scalese S. (2015). Graphene oxide and titania hybrid Nafion membranes for efficient removal of methyl orange dye from water. Carbon.

[19] Bhattacharjee A., Ahmaruzzaman M., Sinha T. (2015). A novel approach for the synthesis of SnO_2_ nanoparticles and its application as a catalyst in the reduction and photodegradation of organic compounds. Spectrochim. Acta Part A Mol. Biomol. Spectrosc..

[20] Kumar M., Mehta A., Mishra A., Singh J., Rawat M., Basu S. (2018). Biosynthesis of tin oxide nanoparticles using Psidium Guajava leave extract for photocatalytic dye degradation under sunlight. Mater. Lett..

[21] Zhu L.-P., Bing N.-C., Yang D.-D., Yang Y., Liao G.-H., Wang L.-J. (2011). Synthesis and photocatalytic properties of core–shell structured α-Fe_2_O_3_@SnO_2_ shuttle-like nanocomposites. Cryst. Eng. Comm..

[22] Oh T. (2018). Effect of double junctions in nano structure oxide materials and gas sensitivity. Trans. Electr. Electron. Mater..

[23] Wang N., Xu J., Guan L. (2011). Synthesis and enhanced photocatalytic activity of tin oxide nanoparticles coated on multi-walled carbon nanotube. Mater. Res. Bull..

[24] Wang C., Wang X., Xu B.-Q., Zhao J., Mai B., Peng P., Sheng G., Fu J. (2014). Enhanced photocatalytic performance of nanosized coupled ZnO/SnO_2_ photocatalysts for methyl orange degradation. J. Photochem. Photobiol. A Chem..

[25] Li Y., Li X., Li J., Yin J. (2006). Photocatalytic degradation of methyl orange by TiO_2_-coated activated carbon and kinetic study. Water Res..

[26] Tian C., Zhang Q., Wu A., Jiang M., Liang Z., Jiang B., Fu H. (2012). Cost-effective large-scale synthesis of ZnO photocatalyst with excellent performance for dye photodegradation. Chem. Commun..

[27] Jiwan S., Prasanna L.L., Yoon-Young C., Jae-Kyu Y., Reddy K.J. (2017). Degradation and mechanism of methyl orange by nanometallic particles under a fenton-like process. Environ. Eng. Sci..

[28] Singh J., Chang Y.-Y., Koduru J.R., Yang J.-K., Singh D.P. (2017). Rapid Fenton-like degradation of methyl orange by ultrasonically dispersed nano-metallic particles. Environ. Eng. Res..

[29] Tammina S.K., Mandal B.K., Kadiyala N.K. (2018). Photocatalytic degradation of methylene blue dye by nonconventional synthesized SnO_2_ nanoparticles. Environ. Nanotechnol. Monit. Manag..

[30] Dai S., Yao Z. (2012). Synthesis of flower-like SnO_2_ single crystals and its enhanced photocatalytic activity. Appl. Surf. Sci..

[31] Pal M., Bera S., Jana S. (2015). Sol–gel based simonkolleite nanopetals with SnO_2_ nanoparticles in graphite-like amorphous carbon as an efficient and reusable photocatalyst. RSC Adv..

[32] Hejazi Juybari S.A., Milani Moghaddam H. (2018). Facile fabrication of porous hierarchical SnO_2_ via a self-degraded template and their remarkable photocatalytic performance. Appl. Surf. Sci..

[33] Dhanalakshmi M., Saravanakumar K., Lakshmi Prabavathi S., Abinaya M., Muthuraj V. (2018). Fabrication of novel surface plasmon resonance induced visible light driven iridium decorated SnO_2_ nanorods for degradation of organic contaminants. J. Alloys. Compd..

[34] Zhang P., Wang L., Zhang X., Shao C., Hu J., Shao G. (2015). SnO_2_-core carbon-shell composite nanotubes with enhanced photocurrent and photocatalytic performance. Appl. Catal. B Environ..

[35] Liang Y., Wu W., Wang P., Liou S.-C., Liu D., Ehrman S.H. (2018). Scalable fabrication of SnO_2_/eo-GO nanocomposites for the photoreduction of CO_2_ to CH_4_. Nano Res..

[36] Begum S., Ahmaruzzaman M. (2018). Biogenic synthesis of SnO_2_/activated carbon nanocomposite and its application as photocatalyst in the degradation of naproxen. Appl. Surf. Sci..

[37] Ding S.-S., Huang W.-Q., Zhou B.-X., Peng P., Hu W.-Y., Long M.-Q., Huang G.-F. (2017). The mechanism of enhanced photocatalytic activity of SnO_2_ through fullerene modification. Curr. Appl. Phys..

[38] Hung-Low F., Ramirez D.A., Peterson G.R., Hikal W.M., Hope-Weeks L.J. (2016). Development of a carbon-supported Sn–SnO_2_ photocatalyst by a new hybridized sol-gel/dextran approach. RSC Adv..

[39] Chen Y., Li H., Ma Q., Zhang Z., Wang J., Wang G., Che Q., Yang P. (2017). A novel electrospun approach for highly-dispersed carambola-like SnO_2_/C composite microparticles with superior photocatalytic performance. Mater. Lett..

[40] Mahadik D.B., Lee Y.K., Park C.-S., Chung H.-Y., Hong M.-H., Jung H.-N.-R., Han W., Park H.-H. (2015). Effect of water ethanol solvents mixture on textural and gas sensing properties of tin oxide prepared using epoxide-assisted sol-gel process and dried at ambient pressure. Solid State Sci..

[41] Jung H.-N.-R., Parale V.G., Kim T., Cho H.H., Park H.-H. (2018). Zirconia-based alumina compound aerogels with enhanced mesopore structure. Ceram. Int..

[42] Parale V.G., Jung H.-N.-R., Han W., Lee K.-Y., Mahadik D.B., Cho H.H., Park H.-H. (2017). Improvement in the high temperature thermal insulation performance of Y_2_O_3_ opacified silica aerogels. J. Alloy. Compd..

[43] Huang R., Hou L., Zhou B., Zhao Q., Ren S. (2005). Formation and characterization of tin oxide aerogel derived from sol-gel process based on Tetra(n-butoxy)tin(IV). J. Non-Cryst. Solids.

[44] Zhao Z., Chen D., Jiao X. (2007). Zirconia aerogels with high surface area derived from sols prepared by electrolyzing zirconium oxychloride solution:  Comparison of aerogels prepared by freeze-drying and supercritical CO_2_(I) Extraction. J. Phys. Chem. C.

[45] Kido Y., Nakanishi K., Miyasaka A., Kanamori K. (2012). Synthesis of monolithic hierarchically porous iron-based xerogels from Iron(III) salts via an epoxide-mediated sol-gel process. Chem. Mater..

[46] Baumann T.F., Kucheyev S.O., Gash A.E., Satcher J.H. (2005). Facile synthesis of a crystalline, high-surface-area SnO_2_ aerogel. Adv. Mater..

[47] Rakibuddin M., Ananthakrishnan R. (2016). A novel Ag deposited nanocoordination polymer derived porous SnO_2_/NiO heteronanostructure for the enhanced photocatalytic reduction of Cr(VI) under visible light. New J. Chem..

[48] Wang Z.-l., Xu D., Huang Y., Wu Z., Wang L.-m., Zhang X.-B. (2012). Facile, mild and fast thermal-decomposition reduction of graphene oxide in air and its application in high-performance lithium batteries. Chem. Commun..

[49] Da Cunha C.R., Toffolo G.H., dos Santos C.E.I., Pezzi R.P. (2013). Structural, optical and chemical characterizations of sol-gel grown tin oxide aerogels. J. Non-Cryst. Solids.

[50] Zhang H., Lv X., Li Y., Wang Y., Li J. (2010). P25-Graphene composite as a high performance photocatalyst. ACS Nano.

[51] Wan F., Lü H.-Y., Wu X.-L., Yan X., Guo J.-Z., Zhang J.-P., Wang G., Han D.-X., Niu L. (2016). Do the bridging oxygen bonds between active Sn nanodots and graphene improve the Li-storage properties?. Energy Storage Mater..

[52] Chen F.-J., Cao Y.-L., Jia D.-Z. (2013). A room-temperature solid-state route for the synthesis of graphene oxide–metal sulfide composites with excellent photocatalytic activity. Cryst. Eng. Comm..

[53] Wang C., Shao C., Zhang X., Liu Y. (2009). SnO_2_ nanostructures-TiO_2_ nanofibers heterostructures: Controlled fabrication and high photocatalytic properties. Inorg. Chem..

[54] Wang P., Zhan S., Xia Y., Ma S., Zhou Q., Li Y. (2017). The fundamental role and mechanism of reduced graphene oxide in rGO/Pt-TiO_2_ nanocomposite for high-performance photocatalytic water splitting. Appl. Catal. B Environ..

[55] Cao Y., Li Y., Jia D., Xie J. (2014). Solid-state synthesis of SnO_2_—Graphene nanocomposite for photocatalysis and formaldehyde gas sensing. RSC Adv..

[56] Zhu Y., Wang Y., Yao W., Zong R., Zhu Y. (2015). New insights into the relationship between photocatalytic activity and TiO_2_–GR composites. RSC Adv..

[57] Yadav H.M., Kim J.-S. (2016). Solvothermal synthesis of anatase TiO_2_—Graphene oxide nanocomposites and their photocatalytic performance. J. Alloy. Compd..

[58] Jo W.-K., Won Y., Hwang I., Tayade R.J. (2014). Enhanced photocatalytic degradation of aqueous nitrobenzene using graphitic carbon–TiO_2_ composites. Ind. Eng. Chem. Res..

